# Características imagenológicas de la osteomielitis de los maxilares evaluada con diferentes métodos diagnósticos. Una revisión

**DOI:** 10.21142/2523-2754-0903-2021-077

**Published:** 2021-10-06

**Authors:** Dirce Fernanda Díaz-Castellón, Jhoana Mercedes Llaguno-Rubio, Paola Eliana Medina-Ocampo

**Affiliations:** 1 Facultad de Odontología de la Universidad San Francisco Xavier de Chuquisaca. Sucre, Bolivia. dircediazcastellon4@gmail.com Facultad de Odontología Universidad San Francisco Xavier de Chuquisaca Sucre Bolivia dircediazcastellon4@gmail.com; 2 División de Radiología Bucal y Maxilofacial de la Universidad Científica del Sur. Lima, Perú. academico@ilaeperu.com, paomedinaocampo@gmail.com Universidad Científica del Sur División de Radiología Bucal y Maxilofacial Universidad Científica del Sur Lima Peru academico@ilaeperu.com paomedinaocampo@gmail.com

**Keywords:** exámenes imagenológicos, osteomielitis maxilar, tomografía computarizada de haz cónico, imaging examinations, osteomyelitis maxillae, cone beam computed tomography

## Abstract

La osteomielitis de los maxilares (OM) es una patología infecciosa que compromete a la médula ósea debido a diversas etiologías, principalmente la odontogénica. El diagnóstico de la OM es un reto para el odontólogo, que se basa en la clínica y los exámenes imagenológicos. La radiografía panorámica es el examen imagenológico que se emplea con mayor frecuencia, pero esta tiene algunas limitaciones que han sido mejoradas con la aparición de nuevos métodos imagenológicos, que incluyen la tomografía computarizada (TC), la tomografía computarizada de haz cónico (TCHC), la imagen por resonancia magnética (IRM) y la gammagrafía ósea con radionúclidos. El propósito de esta revisión fue describir las características imagenológicas de la osteomielitis de los maxilares de acuerdo con todos los métodos de diagnóstico radiológico disponibles según la literatura contemporánea, que permitirán al radiólogo tener más conocimiento y mejorar sus informes en la práctica diaria.

## Abstract (en)

Osteomyelitis of the jaws (OM) is an infectious pathology involving the bone marrow. It is due to various etiologies, the main one being odontogenic. The diagnosis of OM is a challenge for dentists, who must rely on clinical and imaging examinations. Panoramic radiography is the most frequently used imaging test, but it has some limitations that have improved with the incorporation of new imaging methods such as computed tomography, cone beam computed tomography, magnetic resonance imaging, and bone scintigraphy with radionuclides. The purpose of this review was to describe the imaging characteristics of OM of the jaws according to the radiological diagnostic methods available in the literature in order to provide radiologisst with greater knowledge and improve reporting in daily practice.

## INTRODUCCIÓN

La osteomielitis es un proceso inflamatorio que afecta a la médula ósea y puede estar presente en los huesos del cráneo, la cara y demás estructuras óseas ^1^. Generalmente, comienza como una infección de la cavidad medular, afecta rápidamente a los sistemas haversianos y se extiende por el periostio del área ^2^^,^^3^. 

La etiopatogenia es multifactorial y su causa principal es la infección por un foco bacteriano, microorganismos (grampositivos, gramnegativos) y, a veces, presencia fúngica ^1^^,^^3^^,^^4^. Otras causas que contribuyen al desarrollo de la patología son los traumas, las fracturas, las enfermedades odontogénicas infecciosas, ciertos fármacos y la radioterapia ^4^^,^^5^. 

La diseminación de la osteomielitis en el maxilar se produce con mucha facilidad, y afecta con mayor frecuencia al sexo masculino, principalmente a la edad promedio de 40 años y en individuos con enfermedades sistémicas. La zona de los molares mandibulares es la más afectada, debido a la vascularización y la calidad del tejido óseo ^6^^-^^8^. La presencia de la patología continúa pese a los procedimientos de higiene oral, el uso de distintas generaciones de antibióticos, el consumo de ciertos medicamentos como los bifosfonatos, entre otros, que incluso hacen que la patología se desarrolle con mayor facilidad ^7^^,^^9^^-^^11^. 

Existen varios sistemas de clasificación de la osteomielitis; el más aceptado es el que se determina de acuerdo con la duración: aguda y crónica. Esto se refleja con diferentes formas y características tanto en la clínica como en las imágenes diagnósticas, lo que causa confusión en los odontólogos y los radiólogos orales ^3^^,^^7^^-^^9^^,^^12^. El diagnóstico diferencial de la osteomielitis incluye infecciones, tumores óseos benignos, neoplasias malignas, trastornos metabólicos, traumatismos y osteonecrosis ^4^^,^^12^^,^^13^. 

Las imágenes desempeñan un rol fundamental en la identificación y detección de la patología, por lo que se toman en cuenta diferentes métodos para el diagnóstico idóneo, entre ellos las radiografías convencionales, la tomografía computarizada (TC), la tomografía computarizada de haz cónico (TCHC), la imagen por resonancia magnética (IRM), la gammagrafía ósea con radionúclidos y las biopsias óseas, que están disponibles para los clínicos y radiólogos; sin embargo, el desconocimiento de las características e indicaciones de cada uno pueden generar confusiones e, incluso, producir conflictos o complicaciones en el tratamiento de la patología ^1^^,^^4^^,^^5^^,^^7^^,^^10^^,^^14^.

El propósito de la presente investigación fue describir las características imagenológicas de la osteomielitis de los maxilares, de acuerdo con los diferentes estudios por imágenes de utilidad para el diagnóstico de esta patología, lo que ayuda a su reconocimiento, así como a los diagnósticos diferenciales.

## MATERIALES Y MÉTODOS

Se realizó una búsqueda bibliográfica sistematizada en artículos referentes al tema publicados hasta marzo del 2021, también se revisaron fuentes de información incluyendo PubMed, SciELO, Google Scholar, a través de las palabras claves (osteomielitis maxilar, infección ósea maxilar, características imagenológicas, tomografía cone-beam). Se obtuvieron 108 artículos en la búsqueda, pero solo se seleccionaron 49, al considerar los más relevantes en función de los criterios de revisión. Se incluyeron artículos originales, revisiones e informes de casos. Se excluyeron artículos de opinión y cartas al editor. La búsqueda bibliográfica priorizó artículos escritos en inglés y español.

### Fisiopatología de la osteomielitis de los maxilares

En el esqueleto craneofacial, así como en todo el sistema óseo, existen entidades responsables del equilibrio altamente dinámico entre la reabsorción y la formación de matriz ósea en el hueso, que son los osteoclastos y osteoblastos, cuyo equilibrio se verá afectado con los elementos y población celular de la inflamación de médula ósea ^14^^,^^15^. 

La osteomielitis ocurre como resultado de una infección generalmente odontogénica, la cual se expande a través de la médula ósea del maxilar y el periostio, lo que produce como respuesta del organismo un proceso inflamatorio que conduce a la hiperemia, que es un aumento del flujo sanguíneo y leucocitos en el área afectada. La pus, que es el cúmulo de bacterias y desechos celulares que no pueden eliminarse mediante los mecanismos de defensa naturales del cuerpo, invade la médula ósea, lo cual crea una presión intramedular alta, que disminuye aún más el flujo sanguíneo a los huesos de la mandíbula. Este estrés en la irrigación sanguínea provoca la pérdida de vitalidad de los elementos del hueso en la parte afectada. La infección corre a través del sistema de Havers y los canales de Volkmann, para extenderse por los huesos medular y cortical, y a medida que el pus perfora el hueso cortical se acumula debajo del periostio, después de algún tiempo. Finalmente, la purulencia sale de los tejidos blandos a través de fístulas intraorales o extraorales ^1^^,^^16^. 

La clasificación de la osteomielitis más aceptada es la que toma en cuenta el tiempo de evolución, es decir, aguda o crónica. La osteomielitis aguda tiene un tiempo de evolución corto, de días o pocas semanas, y solo en ocasiones la pérdida ósea es evidente radiográficamente. Se puede subdividir en formas supurativas y no supurativas, así como en formas hematógenas. La osteomielitis crónica, en cambio, es recurrente y persistente, se extiende de meses a años, y puede clasificarse, según el agente causal, en formas supurativas, no supurativas y esclerosantes. 

La osteomielitis crónica es una afección grave que requiere tratamiento intrahospitalario inmediato, antibióticos y cirugía. Radiográficamente, se pueden observar imágenes difusas mal definidas, patrones óseos trabeculares radiopacos, radiolúcidos y mixtos, presencia de hueso necrótico (secuestro óseo), aposición de hueso nuevo, tractos fistulosos y reacción periostal ^17^. 

Otro sistema de clasificación divide la osteomielitis crónica en osteomielitis supurativa, osteonecrosis de la mandíbula y osteonecrosis de la mandíbula relacionada con bifosfonatos. La osteomielitis crónica no supurativa de la mandíbula es conocida también en la literatura como osteomielitis crónica primaria, osteomielitis esclerosante difusa, osteomielitis de Garré y osteomielitis crónica mandibular juvenil ^18^^-^^20^. 

### Características imagenológicas de la osteomielitis de los maxilares 

Existen diferentes métodos de ayuda diagnóstica de la osteomielitis, cuya elección debe estar en función de cada etapa de la enfermedad, lo que hace necesario el conocimiento de las características imagenológicas, ya que el diagnóstico definitivo se confirmará mediante la correlación clínico-radiológica y el examen histopatológico ^21^^,^^22^. 

Entre los métodos disponibles para la evaluación están las radiografías simples, la TC, la TCHC, las IRM y las imágenes nucleares con sustancias de contraste como la gammagrafía, todos ellos utilizados para diagnosticar la enfermedad de acuerdo con parámetros que reflejan la destrucción ósea, teniendo en cuenta que el aspecto radiológico de la osteomielitis varía considerablemente según el tipo de respuesta inflamatoria subyacente, la edad y las condiciones del paciente ^19^^,^^22^^,^^23^. 

En la radiografía panorámica, los hallazgos radiográficos de la osteomielitis de los maxilares incluyen la apariencia de hueso apolillado, imágenes radiolúcidas esféricas u ovoides con una esclerosis, mala definición, expansión ósea y reacción perióstica (piel de cebolla), debido a la formación nueva de periostio. Se observa en ocasiones la presencia de secuestros óseos vistos como imágenes radiopacas dentro del área de destrucción ósea; sin embargo, en una etapa temprana, de cuatro a ocho semanas, no se observan grandes signos radiográficos, lo que lleva a confundirla con otra entidad. En general, la información que brinda la radiografía panorámica es limitada debido a la superposición, que dificulta el análisis preciso de la lesión ^5^^,^^23^^-^^25^. 

Respecto de la TCHC, las características imagenológicas involucran la pérdida del patrón trabecular con tractos de resorción radiolúcidos redondos internos, expansión mínima de las tablas óseas, reducción de la corteza alveolar, borde esclerótico periférico y estratificación cortical (involucro). Hay reportes en la literatura en las que se observa la presencia de un lecho alveolar cercano difuso, parcialmente corticalizado, el conducto dentario inferior con la cortical difusa o con pérdida de esta ^26^. 

En esta técnica se identifican distintos patrones óseos. El mixto es el más común, en el que la osteoesclerosis se combina con la osteólisis; otro es el patrón esclerótico y hay otro menos común, que presenta secuestros óseos ^21^. La TCHC es la herramienta auxiliar específica para tejidos duros de cabeza y cuello, pues disminuye la radiación en comparación con la TC. Además, brinda gran precisión para la evaluación del tejido duro, lo que ayuda a detectar la patología en etapas más tempranas, monitorear el desarrollo patológico y también a la planificación quirúrgica de la patología. En general, las características imagenológicas son mejores que las obtenidas con métodos bidimensionales ^20^^,^^27^. 

Por otro lado, la TC permite la evaluación de la cortical ósea, ancho óseo alterado, secuestros, fracturas patológicas, extensión de las esclerosis, localización de fistulas, la relación con estructuras anatómicas próximas a la lesión, la ubicación de defectos trabeculares y la reacción perióstica; pero la radiación es mucho mayor que en la TCHC y los contrastes de tejidos duros del esqueleto craneofacial en esta son inadecuados, lo cual que la técnica se complemente con radiografías extrabucales o intrabucales para aumentar la precisión y definición de la técnica. El empleo de la TC es de mayor utilidad cuando se combina con la medicina nuclear para la detección de la radiación gamma ^21^^,^^28^^,^^29^. También se usa la TC por emisión de fotón único (SPECT) y la TC por emisión de positrones (PET). Con respecto al SPECT, la osteomielitis y la osteonecrosis son sensibles, por lo que obtienen imágenes metabólicas y morfológicas; para ello se emplea un radiofármaco diseminado por vía sanguínea hacía el área de estudio. La técnica permite que la patología sea identificable en una etapa más temprana, en la que los cambios metabólicos sean evidentes, el foco de la patología y su diseminación sean observables, lo que ayuda al tratamiento temprano. También es empleada para el seguimiento posquirúrgico, pero su resolución y especificidad es inferior a la del TC-PET ^30^^,^^31^. 

La técnica por emisión de positrones PET usa radiofármacos que actúan a nivel molecular, se puede combinar con TC e IMR. El PET-TC tiene como resultado imágenes con mayor contraste, mejor resolución espacial y localización exacta en comparación con otras técnicas, como las imágenes bidimensionales y las tomografías. El tiempo de exposición de la técnica es más corto. Esta registra focos o señales de todo el cuerpo, donde la captación de glucosa y granulocitos es mayor, alrededor de la lesión, permite obtener imágenes en las que sea posible evaluar la presencia de una enfermedad crónica, alteraciones anatómicas no evidentes, la respuesta al tratamiento de la patología y la precisión de las lesiones inflamatorias focales ^30^. La especificidad y precisión de la técnica es primordial en la identificación de patologías en etapas tempranas. Sin embargo, se debe evaluar su costo-beneficio. Las imágenes nucleares solo dan parámetros sustanciales de la lisis ósea, por lo que siempre se debe tener una correlación de la clínica en conjunto con los exámenes auxiliares ^30^^,^^32^. 

La IRM proporciona detalle de tejidos blandos relacionados con la lesión del hueso medular y demuestra claramente la celulitis; sin embargo, el especialista en imágenes debe tener experiencia para evitar errores en su interpretación y confundirla con cambios fisiológicos, como un edema óseo. La irradiación de esta técnica es nula, por lo que es adecuada para pacientes jóvenes y con contraindicaciones a la irradiación ^32^^,^^33^. 

### Diagnósticos diferenciales de la osteomielitis de los maxilares de acuerdo con sus características imagenológicas

Los hallazgos de imágenes son muy sugestivos y pueden confundir al clínico. Dentro de los diagnósticos diferenciales más frecuentes están las infecciones odontogénicas, donde las imágenes radiolúcidas por la osteólisis son evidentes; por esto, es primordial la clínica de las lesiones inflamatorias y un estudio que detalle mucho mejor el tejido afectado ^33^^,^^34^. 

Las displasias fibro-óseas no neoplásicas son entidades que, por las características imagenológicas y la radiopacidad con zonas radiolúcidas difusas, pueden llevar a tener un diagnóstico controversial de la patología. Por lo tanto, se recomienda el empleo de la TCHC que permite evaluar mejor las características de la imagen, la expansión ósea, la relación con estructuras adyacentes y su morfología, lo cual permite realizar un diagnóstico diferencial. El diagnóstico de estas entidades son un reto para el clínico, porque lo lleva a realizar una historia clínica minuciosa, estudios por imágenes y, muchas veces, análisis histopatológicos ^32^^,^^35^^-^^37^. 

El tumor óseo benigno osteoblastoma es otra entidad que radiográficamente se muestra como una imagen mixta, mal definida con destrucción de la cortical. En su etapa inicial puede producir una confusión a la hora del diagnóstico, por lo que la literatura propone el empleo de la IRM como la técnica auxiliar, ya que logra identificar zonas hiperintensas e hipointensas que están presentes con mayor frecuencia en las alteraciones óseas ^32^^,^^37^^,^^38^. 

Las lesiones líticas, como los sarcomas a nivel óseo, se presentan como imágenes radiolúcidas, radiopacas y mixtas, dependiendo del nivel de osificación. La similitud en algunas características de la osteomielitis maxilar es evidente, como en el sarcoma de Ewing, en el cual se evidencia imagenológicamente la reacción periostal (capas de cebolla), similar a la característica presente en la osteomielitis maxilar. El empleo de la IRM en estas entidades permite que el estudio de las partes blandas de la lesión sea más minucioso ^39^^,^^40^. 

La osteonecrosis tiene características radiográficas como la radiolucidez mal definida, la esclerosis ósea y los secuestros, que se asemejan a la imagen que presenta la osteomielitis. Estas patologías muestran similitudes imagenológicas y clínicas estrechas, lo que lleva a realizar un estudio histopatológico que hace posible diferenciar las patologías, lo que ayudará a resolver el enigma del diagnóstico presuntivo. La osteonecrosis, como presenta bastante similitud con la osteomielitis en los maxilares, requiere de una buena historia clínica y de estudios imagenológicos adecuados que permitan llegar a un correcto diagnóstico ^22^^,^^23^^,^^41^^-^^43^. 

## DISCUSIÓN

La osteomielitis de los maxilares es una inflamación ósea que compromete el periostio, el cortical y la médula ósea. Su presencia se debe a diferentes factores, uno de la más comunes es la infección odontogénica, el cual se observa con mayor frecuencia en varones, en proporción de 5:1 respecto de las mujeres ^1^^,^^7^^,^^44^^,^^45^. 

Las formas de presentación de la osteomielitis de los maxilares tienen características clínicas y radiológicas diferentes que deben ser conocidas por el clínico y el radiólogo a la hora de emitir un diagnóstico y aplicar un tratamiento apropiado. Los exámenes con los que se cuenta van desde aquellos en los que se puede analizar la lesión en dos dimensiones, como la radiografía panorámica, hasta imágenes por tomografía computarizada, la resonancia magnética, la medicina nuclear y los estudios histopatológicos, entre los más conocidos. La apariencia de hueso apolillado, esclerosis ósea, radiolucidez mal definida, secuestros y otras características, que son las más comunes, generan en el clínico confusión con otras patologías que muestran características imagenológicas similares, por lo que se precisará de otro tipo de examen que brinde características más definidas y que ayude a obtener un diagnóstico, tratamiento, prevención y seguimiento adecuados. Por ello, se hace necesario conocer qué características imagenológicas de la osteomielitis de los maxilares están presentes en los diferentes métodos diagnósticos, los cuales puedan complementar la historia de la patología.

Los métodos actuales logran identificar a la osteomielitis maxilar en etapas tempranas, intervenir en ella, delimitar con precisión la extensión y analizar el comportamiento de la lesión; además, se logra reducir la radiación en aquellos pacientes contraindicados. Sin embargo, los estudios con los que se cuentan muchas veces no son accesibles por el costo, por ello solo se reportan casos en estadios crónicos, ya que la osteomielitis en etapas tempranas, en el caso de las imágenes bidimensionales, puede ser confundida con lesiones óseas menos complicadas, como una periodontitis crónica ^46^. 

La literatura propone la TCHC como el mejor método para la planificación quirúrgica de una osteomielitis, al ser comparada con la imagen en dos dimensiones donde se contaba con bastantes interposiciones ^8^^,^^9^^,^^30^^,^^32^^,^^33^. La identificación temprana y el seguimiento de la osteomielitis maxilar es posible con la medicina nuclear PET/TCHC ^30^^,^^34^^,^^46^^,^^47^. La IRM en la osteomielitis maxilar, además de analizar al detalle tejidos blandos en la lesión, se convierte la primera opción para pacientes con restricción a la radiación y en pacientes jóvenes ^15^^,^^34^^,^^48^. 

Las imágenes mixtas, con bordes irregulares y mal definidos, y con presencia de uno o varios focos radiopacos, son características de la osteomielitis maxilar , por lo que puede ser confundida con otras entidades patológicas, como displasias fibro-óseas no neoplásicas, que producen dudas al momento de emitir un diagnóstico. Esto lleva a requerir la historia clínica del paciente, los estudios imagenológicos y el examen histopatológico para tener un diagnóstico preciso ^22^^,^^35^^,^^48^. 

Los radiólogos deben considerar actualmente el uso de imágenes de tres dimensiones, como la tomografía computarizada, para un diagnóstico preciso e idóneo de los problemas de osteomielitis. Localizar la lesión con una resolución mejorada hará que su reconocimiento sea más preciso a la hora de realizar el tratamiento.

El presente estudio tuvo algunas limitaciones. Una de ellas fue que la búsqueda de la información en la literatura presentó mayoritariamente estudios realizados en imágenes bidimensionales y con complicaciones de consumo de bifosfonatos, aunque se pudo incluir información contemporánea de estudios con TCHC. Otra de las limitantes fue la escasa y dispersa información referente a los diagnósticos diferenciales en osteomielitis maxilar, por lo que futuros estudios deberían enfocarse en este punto.

Finalmente, en este estudio se revisaron la fisiopatología, las características imagenológicas de la osteomielitis de los maxilares y el diagnóstico diferencial de la patología, que puede ser de utilidad para comprender las características de las imágenes de una osteomielitis diferenciarla, reconocerla y contribuir a su diagnóstico precoz. 

## CONCLUSIONES

Los radiólogos necesitan reconocer las características imagenológicas de la osteomielitis como puntos diferenciales entre patologías óseas que tienen similitudes imagenológicas para evitar diagnósticos equivocados y tratamientos erróneos. La participación del radiólogo en la guía del tratamiento y seguimiento de estos pacientes es fundamental. Los métodos auxiliares imagenológicos actuales de la osteomielitis maxilar son indispensables para el tratamiento, la planificación quirúrgica, el seguimiento de la patología y su prevención.

## Fuente de financiamiento:

Autofinanciado

## References

[1] Mardini S, Gohel A (2018). Imaging of Odontogenic Infections. Radiol Clin North Am.

[2] Schmitt SK (2017). Osteomyelitis. Infect Dis Clin North Am.

[3] Sood R, Gamit M, Shah N, Mansuri Y, Naria G (2020). Maxillofacial Osteomyelitis in immunocompromised patients A demographic retrospective study. J Maxillofac Oral Surg.

[4] Kusuyama Y, Matsumoto K, Okada S, Wakabayashi K, Takeuchi N, Yura Y (2013). Rapidly progressing osteomyelitis of the mandible. Case Rep Dent.

[5] Mehra H, Gupta S, Gupta H, Sinha V, Singh J (2013). Chronic suppurative osteomyelitis of mandible a case report. Craniomaxillofac Trauma Reconstr.

[6] Julien Saint Amand M, Sigaux N, Gleizal A, Bouletreau P, Breton P (2017). Chronic osteomyelitis of the mandible A comparative study of 10 cases with primary chronic osteomyelitis and 12 cases with secondary chronic osteomyelitis. J Stomatol Oral Maxillofac Surg.

[7] Sadaksharam J, Murugesan M (2019). Osteomyelitis of Maxilla A rare finding from a radiologist point of view. Contemp Clin Dent.

[8] Fullmer JM, Scarfe WC, Kushner GM, Alpert B, Farman AG (2007). Cone beam computed tomographic findings in refractory chronic suppurative osteomyelitis of the mandible. Br J Oral Maxillofac Surg.

[9] Park MS, Eo MY, Myoung H, Kim SM, Lee JH (2019). Early diagnosis of jaw osteomyelitis by easy digitalized panoramic analysis. Maxillofac Plast Reconstr Surg.

[10] Bocchialini G, Ferrari L, Rossini M, Bozzola A, Burlini D (2017). Chronic nonbacterial osteomyelitis involving the mandible A case report. Int J Surg Case Rep.

[11] Trivellato AE, Ribeiro MC, Sverzut CE, Bonucci E, Nanci A, de Oliveira PT (2009). Osteopetrosis complicated by osteomyelitis of the maxilla and mandible light and electron microscopic findings. Head Neck Pathol.

[12] Andre CV, Khonsari RH, Ernenwein D, Goudot P, Ruhin B (2017). Osteomyelitis of the jaws A retrospective series of 40 patients. J Stomatol Oral Maxillofac Surg.

[13] Mandell JC, Khurana B, Smith JT, Czuczman GJ, Ghazikhanian V, Smith SE (2018). Osteomyelitis of the lower extremity pathophysiology, imaging, and classification, with an emphasis on diabetic foot infection. Emerg Radiol.

[14] Taddio A, Ferrara G, Insalaco A, Pardeo M, Gregori M, Finetti M (2017). Dealing with Chronic Non-Bacterial Osteomyelitis a practical approach. Pediatr Rheumatol Online J.

[15] Camison L, Mai RS, Goldstein JA, Costello BJ, Torok KS, Losee JE (2018). Chronic recurrent multifocal osteomyelitis of the mandible A diagnostic challenge. Plast Reconstr Surg.

[16] Monsour PA, Dalton JB (2010). Chronic recurrent multifocal osteomyelitis involving the mandible case reports and review of the literature. Dentomaxillofac Radiol.

[17] Picnus DJ (2009). Osteomielitis del esqueleto craneofacial. Semin Plast.

[18] Rajkumar GC, Hemalatha M, Shashikala R, Kumar DV (2010). Recurrent chronic suppurative osteomyelitis of the mandible. Indian J Dent Res.

[19] Koorbusch GF, Deatherage JR, Cure JK (2011). How can we diagnose and treat osteomyelitis of the jaws as early as possible. Oral Maxillofac Surg Clin North Am.

[20] Dym H, Zeidan J (2017). Microbiology of acute and chronic osteomyelitis and antibiotic treatment. Dent Clin North Am.

[21] Garcia CM, Garcia MA, Garcia RG, Gil FM (2013). Osteomyelitis of the mandible in a patient with osteopetrosis Case report and review of the literature. J Maxillofac Oral Surg.

[22] Anesi A, Generali L, Sandoni L, Pozzi S, Grande A (2019). From osteoclast differentiation to osteonecrosis of the jaw: molecular and clinical insights. Int J Mol Sci.

[23] De Antoni CC, Matsumoto MA, Silva AAD, Curi MM, Santiago JF, Sassi LM (2018). Medication-related osteonecrosis of the jaw, osteoradionecrosis, and osteomyelitis A comparative histopathological study. Braz Oral Res.

[24] Sun YK, Cha JK, Thoma DS, Yoon SR, Lee JS, Choi SH (2018). Bone regeneration of peri-implant defects using a collagen membrane as a carrier for recombinant human bone morphogenetic protein-2. Biomed Res Int.

[25] Marx RE, Tursun R (2013). Response to - a commentary on &raquo;,» &reg;,® &sect;,§ &shy;,­ &sup1;,¹ &sup2;,² &sup3;,³ &szlig;,ß &THORN;,Þ &thorn;,þ &times;,× &Uacute;,Ú &uacute;,ú &Ucirc;,Û &ucirc;,û &Ugrave;,Ù &ugrave;,ù &uml;,¨ &Uuml;,Ü &uuml;,ü &Yacute;,Ý &yacute;,ý &yen;,¥ &yuml;,ÿ &para;,¶ Suppurative osteomyelitis, bisphosphonate induced 1 osteonecrosis, osteoradionecrosis: a blinded histopathologic comparison and its 2 implications for the mechanism of each disease &raquo;,» &reg;,® &sect;,§ &shy;,­ &sup1;,¹ &sup2;,² &sup3;,³ &szlig;,ß &THORN;,Þ &thorn;,þ &times;,× &Uacute;,Ú &uacute;,ú &Ucirc;,Û &ucirc;,û &Ugrave;,Ù &ugrave;,ù &uml;,¨ &Uuml;,Ü &uuml;,ü &Yacute;,Ý &yacute;,ý &yen;,¥ &yuml;,ÿ &para;,¶ by R.E. Marx and R. Tursun (Int. J. Oral. Maxillofac. Surg. 41 (3) (2012) 283-289). Int J Oral Maxillofac Surg.

[26] Schulze D, Blessmann M, Pohlenz P, Wagner KW, Heiland M (2006). Diagnostic criteria for the detection of mandibular osteomyelitis using cone-beam computed tomography. Dentomaxillofac Radiol.

[27] Marx RE, Tursun R (2012). Suppurative osteomyelitis, bisphosphonate induced osteonecrosis, osteoradionecrosis a blinded histopathologic comparison and its implications for the mechanism of each disease. Int J Oral Maxillofac Surg.

[28] Haeffs TH, Scott CA, Campbell TH, Chen Y, August M (2018). Acute and chronic suppurative osteomyelitis of the jaws A 10-year review and assessment of treatment outcome. J Oral Maxillofac Surg.

[29] Guimaraes EP, Pedreira FR, Jham BC, de Carli ML, Pereira AA, Hanemann JA (2013). Clinical management of suppurative osteomyelitis, bisphosphonate-related osteonecrosis, and osteoradionecrosis report of three cases and review of the literature. Case Rep Dent.

[30] Hakim SG, Bruecker CW, Jacobsen H, Hermes D, Lauer I, Eckerle S (2006). The value of FDG-PET and bone scintigraphy with SPECT in the primary diagnosis and follow-up of patients with chronic osteomyelitis of the mandible. Int J Oral Maxillofac Surg.

[31] Liu D, Zhang J, Li T, Li C, Liu X, Zheng J (2019). Chronic osteomyelitis with proliferative periostitis of the mandibular body report of a case and review of the literature. Ann R Coll Surg Engl.

[32] Fenerty S, Shaw W, Verma R, Syed AB, Kuklani R, Yang J (2017). Florid cemento-osseous dysplasia review of an uncommon fibro-osseous lesion of the jaw with important clinical implications. Skeletal Radiol.

[33] Mupparapu M, Shi KJ, Ko E (2020). Differential diagnosis of periapical radiopacities and radiolucencies. Dent Clin North Am.

[34] Bolouri C, Merwald M, Huellner MW, Veit-Haibach P, Kuttenberger J, Perez-Lago M (2013). Performance of orthopantomography, planar scintigraphy, CT alone and SPECT/CT in patients with suspected osteomyelitis of the jaw. Eur J Nucl Med Mol Imaging.

[35] Bernaola-Paredes WE, Sugaya NN, Bergamini ML, Braz-Silva PH (2020). A distinct fibro-osseous lesion of the jaws affecting the maxilla. J Oral Maxillofac Pathol.

[36] Brody A, Zalatnai A, Csomo K, Belik A, Dobo-Nagy C (2019). Difficulties in the diagnosis of periapical translucencies and in the classification of cemento-osseous dysplasia. BMC Oral Health.

[37] Gonuguntla S, Nama R, Vanajakshi CN, Mandadi SR, Madireddy JR (2020). Osteoblastoma of mandible in child A case report. J Pharm Bioallied Sci.

[38] Limaiem F, Byerly DW, Singh R (2021). Osteoblastoma.En StatPearls.

[39] Jackson TM, Bittman M, Granowetter L (2016). Pediatric malignant bone tumors A review and update on current challenges, and emerging drug targets. Curr Probl Pediatr Adolesc Health Care.

[40] ElKordy MA, ElBaradie TS, ElSebai HI, KhairAlla SM, Amin AAE (2018). Osteosarcoma of the jaw Challenges in the diagnosis and treatment. J Egypt Natl Canc Inst.

[41] Pereira T, Gomes CC, Brennan PA, Fonseca FP, Gomez RS (2019). Fibrous dysplasia of the jaws Integrating molecular pathogenesis with clinical, radiological, and histopathological features. J Oral Pathol Med.

[42] Kishimoto H, Noguchi K, Takaoka K (2019). Novel insight into the management of bisphosphonate-related osteonecrosis of the jaw (BRONJ). Jpn Dent Sci Rev.

[43] Yoneda T, Hagino H, Sugimoto T, Ohta H, Takahashi S, Japanese Allied Committee on Osteonecrosis of the J (2017). Antiresorptive agent-related osteonecrosis of the jaw Position Paper 2017 of the Japanese Allied Committee on Osteonecrosis of the Jaw. J Bone Miner Metab.

[44] Silva BSF, Bueno MR, Yamamoto-Silva FP, Gomez RS, Peters OA, Estrela C (2017). Differential diagnosis and clinical management of periapical radiopaque/hyperdense jaw lesions. Braz Oral Res.

[45] Cavalcante MB, de Oliveira Lima AL, Junior MA, Santos MB (2016). Florid cemento-osseous dysplasia simultaneous the chronic suppurative osteomyelitis in mandible. J Craniofac Surg.

[46] Gaeta-Araujo H, Vanderhaeghen O, Vasconcelos KF, Coucke W, Coropciuc R, Politis C (2021). Osteomyelitis, osteoradionecrosis, or medication-related osteonecrosis of the jaws Can CBCT enhance radiographic diagnosis?. Oral Dis.

[47] Malina-Altzinger J, Klaeser B, Suter VGA, Schriber M, Vollnberg B, Schaller B (2019). Comparative evaluation of SPECT/CT and CBCT in patients with mandibular osteomyelitis and osteonecrosis. Clin Oral Investig.

[48] Bailey DL, Willowson KP (2013). An evidence-based review of quantitative SPECT imaging and potential clinical applications. J Nucl Med.

[49] Omami G (2020). Ossified Mandibular Mass Osteosarcoma. Ear Nose Throat J.

